# An explainable hybrid deep learning framework for precise skin lesion segmentation and multi-class classification

**DOI:** 10.3389/fmed.2025.1681542

**Published:** 2025-10-13

**Authors:** Muhammad Fiaz, Muhammad Bilal Shoaib Khan, Abdul Hannan Khan, Anas Bilal, Monir Abdullah, Abdulbasit A. Darem, Raheem Sarwar

**Affiliations:** ^1^Department of Computer Science, Green International University, Lahore, Pakistan; ^2^Department of Software Engineering, University of Central Punjab, Lahore, Pakistan; ^3^College of Information Science and Technology, Hainan Normal University, Haikou, China; ^4^Department of Computer Science and Artificial Intelligence, College of Computing and Information Technology, University of Bisha, Bisha, Saudi Arabia; ^5^Center for Scientific Research and Entrepreneurship, Northern Border University, Arar, Saudi Arabia; ^6^Department of Computer Science, College of Science, Northern Border University, Arar, Saudi Arabia; ^7^OTEHM, Manchester Metropolitan University, Manchester, United Kingdom

**Keywords:** skin disease, classification, segmentation, explainable AI, Grad-CAM

## Abstract

**Introduction:**

Skin diseases, ranging from benign conditions to malignant tumors such as melanoma, present substantial diagnostic challenges due to their visual complexity and the inherent subjectivity in manual examination.

**Methods:**

This paper introduces a hybrid deep learning framework specifically designed for skin lesion segmentation and multi-class classification using dermoscopic images. The proposed model integrates a dual-task architecture, which combines a U-Net-based segmentation network with a classification module based on the EfficientNet-B0 backbone. To improve model interpretability and foster clinical trust, Grad-CAM is incorporated, allowing clinicians to visualize heatmaps that highlight the regions influencing the model’s decisions.

**Results:**

The model was trained and evaluated on the HAM10000 dataset, demonstrating robust performance, with a Dice coefficient surpassing 0.85 for segmentation and classification accuracy nearing 85%. Despite challenges such as class imbalance and the variety of lesion types, the model provides reliable results across different skin conditions.

**Discussion:**

The use of explainable AI (XAI) enhances transparency, a crucial factor in the clinical acceptance of AI-based diagnostic tools. This approach shows promise in improving diagnostic accuracy and supporting dermatologists, especially in resource-constrained settings, by providing both accurate lesion delineation and reliable class predictions. Future research will focus on improving the model’s generalizability, addressing underrepresented classes, and validating its effectiveness in real-world scenarios.

## 1 Introduction

Skin diseases form a wide and heterogeneous spectrum characterized by a series of dermatologic disorders giving constant challenges in its diagnosis and management (1). Being the largest body part, the skin is the most important physical defense against external agents and at the same time subject to a variety of pathologies (2). The skin disorders comprise conditions like acne, eczema, and psoriasis and malignancies like melanoma (3). The pathologies that lead to these conditions are diverse; they can be genetic predispositions, pathologies of immune system, and other environmental factors like sunlight, and air pollution. An example is acne that is regularly related to the fluctuation of hormones, and eczema can be attributed to allergies or genetics (4, 5). Melanoma in particular, has very close connection with long time exposure to sun and increased cases of it are being reported in areas with high levels of ultraviolet radiation (6, 7). Understanding the determinants could mean accurate diagnosis and effective treatment.

The prevalence of dermatological conditions has been steadily increasing during the last few years and there has been notable expansion in cases of skin diseases recorded both in developed and developing countries (8, 9). Melanoma is still more common in experienced sunny areas (9); however, more and more disorders of the skin, including, eczema, and psoriasis are increasing their distribution, especially in urban big cities where it is possible to find many vertices that promote their increasing rate, including polluted air and stressful living styles. As the incidence increases in multiple groups of people, especially between 18 and 45 years of age (10), there is a marked necessity to enhance diagnostic techniques that can assist the clinicians in providing timely and precise therapy. Clear discrimination and subdivision of skin lesions still require intense professional competence which in the past has been based on hectic manual inspection that is still vulnerable to subjective errors. Consequently, there is an increasing emphasis on machine learning and deep learning techniques as methods to automate and enhance the diagnostic process (11).

The integration of artificial intelligence and deep learning has greatly advanced medical imaging, disease diagnosis, and healthcare decision-making. At the systemic level, innovation networks play a critical role in supporting regional digital health systems and enabling collaboration in advanced medical equipment industries (12). In dermatology, novel architectures such as multimodal masked autoencoders for vitiligo stage classification (13, 14) and interaction transformer modules for white patchy skin lesion analysis (14) have improved diagnostic accuracy. Similarly, transformer-based methods like CenterFormer have enhanced unconstrained dental plaque segmentation (15), while deep learning models have accelerated super-resolution ultrasound microvessel imaging (16). Clinical studies further underscore the importance of AI by complementing retrospective analyses of immunotherapy-induced psoriasis, such as pembrolizumab- and nivolumab-related cases (17, 18). In ophthalmology, adaptive multi-scale feature fusion networks have shown strong potential in diabetic retinopathy classification (19), and bio-inspired optimization algorithms have supported MRI segmentation in sports injury assessment (20). Parallel research in oncology has emphasized efficient melanoma detection using pixel intensity masking (21), hybrid deep learning with dual encoders and channel-wise attention (22), precision-driven dual encoder segmentation models (23), and synergistic CNN-based frameworks for early melanoma detection and treatment strategies (24).

Moreover, Over the past few years, deep learning has achieved remarkable advancements in the realm of medical image analysis, particularly within dermatology Convolution-based neural networks are extensively utilized in these applications, excel at discerning patterned features within images with high accuracy (25–27). The same has made the models essential in automatic identification and demarcation of skin lesions thus ensuring fast and reliable diagnoses (28). The AI systems by providing potential clinical assistance to the diagnosis improve its effectiveness, providing a second opinion, helping diagnosticians see the early symptoms that cannot be noticed by humans, and cutting the time it would take to analyze the images and obtain the findings. Further technological optimization is likely to achieve a rather decisive role of AI in dermatological practice, especially in those areas where the availability of specialized medical staff is restricted.

Despite these advances, existing methods still fall short of clinical demands. Many models lack precision in delineating lesion boundaries, fail to generalize across varied skin pathologies, and suffer from the black-box nature of deep learning frameworks, which limits interpretability and reduces clinician trust (29, 30), which handicaps the clinicians to entrust and incorporate the systems in standard care. However, even though some of the segmentation methods perform well on benchmarking, they always demand very high-frequency images and provide inefficient results on various skin lesions and fail to offer clear insight into their decision-making framework. Powerful segmentation together with Grad-CAM explainable images can considerably drive clinical accuracy and location evaluation of skin lesions (31). Such capability is especially advantageous in settings characterized by scarce dermatological expertise, where diagnostic delays are highest and the demand for quality care is most acute, notably in regions of elevated ultraviolet (UV) exposure or within low- and middle-income countries (LMICs).

In this study, we introduce a multi-task framework that integrates lesion segmentation, multi-class classification, and model explainability within a single pipeline. Unlike many existing approaches that treat segmentation and classification as separate tasks, our design emphasizes their joint optimization, demonstrating that improvements in segmentation can directly enhance classification accuracy, and vice versa. To address the persistent challenge of imbalanced dermatological datasets, the framework incorporates curriculum-based training together with stochastic hard-example replay, a strategy that, to our knowledge, has not yet been explored in this context. Beyond improving diagnostic accuracy, this integrated approach also enhances model interpretability, thereby strengthening both the methodological rigor and the clinical relevance of the proposed system.

### 1.1 Problem statement

Despite advances in dermatological image segmentation, existing methods often fail to meet the critical requirements for real-world clinical deployment. In particular, they struggle to achieve: (i) precise and accurate delineation of lesion boundaries, (ii) steady effectiveness across a diverse array of dermatological conditions and (iii) model interpretability, which is essential for clinicians to trust and effectively use the system. While segmentation models have demonstrated promising outcomes, they frequently struggle to achieve high accuracy across various skin conditions and offer limited transparency in their decision-making processes.

The ability to predict the correct lesion mask, combined with Grad-CAM’s visual explanation, significantly aids dermatologists in precisely recognizing the type and location of the skin disease. These advancements are particularly critical in clinical settings with limited access to skilled dermatologists, as they reduce diagnostic delays and improve the overall quality of care, particularly in areas with significant UV exposure or in low- and middle-income countries (LMICs).

### 1.2 Objectives and approach

The current research study focuses on some of the modern challenges of medical imaging analysis and suggesting a hybrid deep-learning model structure where lesion segmentation and classification are done in the same deep-learning model architecture. In order to sustain high diagnostic performance yet enhance transparency, the procedural design also integrates explainable-AI (XAI) techniques.

The module segmentation was defined as a multi-scale dilated U-Net to ensure contextual information remained despite limiting the complexity of the model. There is then a separate but complementary classification module with cases of tumors being dichotomized into malignant and benign. To interpret the model, heatmaps are generated using Grad-CAM, which mark the sections of the input image that play a role in determining the model’s predictions. Such an integrated approach not only increases the rate of accuracy in classification but also visual explanations that are congruent with clinical expertise.

Combining the high diagnostic accuracies with explanations that can be understood by clinicians, the strategy aims at widening the know-how acceptance of AI-based diagnostic systems, and thus enhancing patient outcomes.

## 2 Literature review

A diagnosis of skin pathology, in particular skin cancer, continues to pose a serious clinical challenge due to the heterogeneity of the lesions at the skin surface and to the apparent visual similarity of different conditions. A broad band of tools has been adopted to additional ascertain that what a dermatologist sees with the help of his eyes and some form of clinical judgment, to cover the extreme (non-invasive to invasive biopsy). However, these conventional methods are known to be biased by the experience of the examiner and therefore bring forth unwanted variability and subjectivity.

The emergence of digital imaging technologies have redefined the scenario with dermoscopy as the most relevant non-surveillance magnification method of cutaneous structures (32). Dermoscopy complements the evaluation of lesions through the increase in visual resolution (33). However, the manual interpretation is slow to die off, retaining its labor-interest nature, and the tendency to produce accidental errors. This has brought about an interest in increasing the technology of computer-aided diagnostic (CAD) system as a potential solution.

The modern advancements of machine learning (and deep learning specifically) have highlighted the possibility of the effective automation of the process of evaluating skin lesions. The use of convolutional neural networks (CNNs) has become the leading approach to the analysis of images in dermatology (34). They are AI-based tools as well, which aim to reduce clinician diagnostic workload, and more importantly improve accuracy with regards to timely melanoma and skin cancer detectives. Empirical evidence, however, indicates that a number of fundamental problems remain such as the need to deal with the issue of class imbalance, issue of model transparency and issue of high generalizability among varying skin conditions. These challenges are vital to success in implementing such systems to the extent of making them reliable and clinically relevant. The following gives an account of recent research studies concerning skin lesions like,

In the current research (35), two convolutional neural networks are studied, namely DenseNet −201 and Inception -V3, and how they can be used to classify cutaneous malignancies. The study was conducted in 2024 and based on the ISIC 2019 archive that includes 25, 331 dermoscopic images, divided into 60, 20, and 20 percent training, validation and testing, respectively. DenseNet-201 was able to achieve general accuracy of 84.3 and Inception V3 hit 81.5. Similar to the efficacy of deep-learning methods to detect skin-lesions, these findings only indicate the fact that the platform still faces significant challenges (including issues of class discrepancy and larger, more thoroughly annotated datasets).

In this study (36), the authors proposed anti-aliasing convolutional neural network (AA -CNN) architecture to supplement classification performance in dermoscopic image, in their 2023 study. The model was assessed in 2022 on the ISIC 2018 archive of 10,015 training and 1,512 test images of seven different categories of skin -lesion. The AA-CNN had an average accuracy and area under receiver operating characteristic of 88.87 and 0.945, respectively, as compared to traditional baseline CNNs. Though the authors do emphasize the benefits of including the use of anti-aliasing filters in order to ensure model robustness, they also note constraints in the matter of generalizability and the complex nature of real-world clinical data.

The authors conduct a study in which they address the question of the usefulness of explainability methods as a way of understanding the decision-making process of skin lesion classifiers (37). The study staff trained an Inception model of −4 with the ISIC 2018 dataset and achieved classification achievement of 89.96–400. They contrasted seven explainability techniques namely Grad-CAM, Score-CAM, LIME, SHAP, ACE, ICE and CME. As previously shown in the analysis, though these techniques indicate pertinent parts of the image or abstract ideas, most of them exhibit uncontrollable artifacts, and, most importantly, most of them do not give viable explanations of model reasoning. Although CME reached the fidelity 88⦻, the study also made a conclusion that neither one method could fully produce satisfactory explanations, which supports the necessity of performing a combination of approaches and being cautious when applying explainable AI in clinical practices.

The current (38) research paper proposes a collaborative deep convolutional neural network, CL-DCNN, a type of neural network that separates the skin lesion into segments and classifies the skin lesion by sharing learning. By using pseudo-label generating, class activation map (CAM), and segmentation mask, the model transforms each of the other, with the aim of performance improvement. The effectiveness of the approach is supported by empirical analyses of the ISIC 2017 and ISIC Archive datasets, where, the Jaccard index would be 79.1 percent for segmentation and the area under the ROC curve is 93.7 percent to classify. These indicators are better than a number of modern techniques. Despite the fact that the proposal operated well on reducing the annotation burdens and enhancing the diagnostic accuracy, its external validity is limited, especially when extended to the real clinical situations.

The authors describe a deep-learning structure adopted in this research (39), which uses a hybrid architecture of a convolutional neural network to enhance the detection of skin lesions. The network was trained with the HAM10000 dataset as a test dataset and the input images were preprocessed, enhanced with data augmentation, and strategies of class -balance were implemented. With evaluation scores, it was found that the proposed model had an accuracy of 94.6 -, which is an improvement compared to various traditional CNN base models including VGG − 16 and ResNet −50. The outcomes validate the advantage of deep-learning methods to analyze dermatological images, but the necessity to consider the problem of the imbalance in the number of processed cases and focus on the model interpretability to stimulate clinical confidence and practical applicability.

In this study (40), the researchers propose an entirely machine-driven Deep Neural Network used to recognize the location of skin lesions and additionally categorize multiclassified dermoscopic therapies, using refined features. They use a 10-layer customized convolutional neural network to perform saliency-guided segmentation and use ResNet101 and DenseNet201 to utilize strong feature extractors. In order to enhance classification, better Moth Flame Optimization (IMFO) algorithm is employed in terms of feature selection and Multiset Maximum Correlation Analysis (MMCA) parameter is used to fuse features. Finally, the final classification is implemented using a Kernel Extreme Learning Machine (KELM) with an accuracy of 90.67 Jennifer Hammond: A Timeline 10,000 delivered Decision-Control OS. Despite the fact that the approach has good performance in both the ISBI and the ISIC data, issues of class imbalance exist, as well as asymmetrical morphology of the lesions.

This study (41) presents an optimized U-Net architecture employing along with pooling operations and attention to support the skin-lesion segmentation allowing the focus thereafter of convolutional neural network (CNN)-supported classifiers. The segmentation subnet performed better, being trained and tested on the ISIC2018 database as it enhanced better boundary delineation and localization of the lesion regions compared to the traditional U-Net. A CNN was trained on the same dataset in the classification task where categorization of seven skin-lesion classes was undertaken, the Dice similarity coefficient was 91.28 percent, and classification accuracy was 89.14 percent. Despite these encouraging statistics, the study is aware of the lingering limitations, such as heterogeneity of lesion sizes and imaging artifacts, which remain the limitations of the overall robustness.

The authors describe a single architecture, called SkinNet, which is U-Net additionally designed to result in the precise distribution of skin lesions (42). This model integrates the dilated convolutions within the bottleneck that seeks to expand the receptive field and dense convolution block to keep spatial detail. Competition on Dice coefficient and Jaccard index for the ISBI 2017 challenge dataset produced 85.1 and a 76.7 index, both above a series of some of the state-of-the-art techniques. These results confirm that SkinNet is resistant to distinguish the lesions of various morphologies and sizes, still, there are complications, especially where the contrast between the lesion and the surrounding skin is low.

The proposed classification framework proposed by the authors in this paper (43) uses the multi-scale convolutional neural network that is tied to the Inception-v 3 architecture. The network was trained using the ISIC 2017 skin lesion dataset taking both coarse and the fine resolution images to obtain both global and local lesion features. Using the training set as a starting point with added images by the ISICMSK21 dataset and various practices to achieve a high level of accuracy which included fine-tuning, image augmentation and 10-model ensembling, led to the development of the resulting classifier producing, on average, 90.3% accurate results with the area-under-the-curve (AUC) of 0.896 with melanoma and 0.990 with seborrhoeic keratosis, respectively. Although this work focuses on the ability of architectural optimizations and careful training scenarios to improve the performance in processing the medical image classification even in a setting that is limited by training data.

In the current research (44), the authors research the effectiveness of deep convolutional neural networks in the detection of skin lesions. The three architectures assessed in the study, namely, AlexNet, ResNet-18, and ResNet-50, are trained using a dataset consisting of 10,015 dermoscopic images of different categories (7). ResNet-50 records the best performance of 86.6 later resnet-18 with the 83.2 accuracy and lastly AlexNet with the performance of 78.4. Such results point to the potential of deep learning models to classify different types of skin lesions. The authors, however, note that there are still challenges that persist and include the availability of either imbalanced data and the need to have a better generalization of features between different classes.

Recent studies have also explored ways to improve fairness and strengthen feature representation. For instance (45), introduced a classification framework that fused deep features to reduce dataset bias, while (46) developed a hybrid model combining InceptionV3 and DenseNet121, which yielded improved classification outcomes. These approaches highlight the importance of feature integration; however, our framework advances this idea further by unifying segmentation, classification, and interpretability within a single pipeline.

### 2.1 Principal contributions

- *Unified dual-task framework:* The proposed work introduces a unified dual-task framework in which a single end-to-end model performs both skin lesion segmentation and multi-class classification. By integrating these two tasks within the same architecture, the network benefits from shared feature representations, which leads to stronger performance than training each task independently
- *Curriculum-guided learning:* To make the learning more stable, the proposed framework establishes curriculum-managed training. In this paradigm model is introduced with other simpler more common samples and then in more complex and less common situations. Graduated exposure of this type reduces the training instability and enhances the ability of the network to address the strong imbalance between the classes of skin lesions dataset.
- *Strong and effective evaluation:* The HAM10000 dataset was carefully evaluated using popular performance measures such as Dice Coefficient, intersection over Union (IoU), accuracy, area under the ROC curve (AUC) and F1 -score. Competitive results were attained with this model, and the inference time per image in this instance was more than 40 milliseconds, which makes it a practical speed (enough) to operate in diagnostic scenarios in real-time.
- *Accurate mask prediction:* In addition to class prediction, each class produces a lesion mask at a high level of precision, allowing proper boundary drawing. This feature guides clinicians in such important decision-making during excision or biopsy planning.
- *Class-aware explanations:* The interpretability is going to be addressed by deploying Grad-CAM into the classification module. The produced heatmaps are class specific visual explanations which can immediately compare the lesion delimitations generated through the segmentation task. This dual view provides extra confidence in clinical practice as it is guaranteed that the areas on which the model predictions are dependent are related to real-life locations of pathology.
- *Class-specific mask generation:* At last, the model produces matching segmentation masks per each of the classes, which will provide it with dual functionality and thus enable dermatologists to spot the type of lesion and its exact location to optimize the diagnostic process and treatment plans.
- *Comprehensive model evaluation:* The present work shows that the considerable performance is generalizable along many clinical lesion types using rigorous validation based upon several performance indicators, namely accuracy, AUC-ROC, and specificity. The model identifies specific competence in the identification of melanoma, basal cell carcinoma or vascular lesions.
- *Practical clinical utility:* In a practical clinical standpoint, the insertion of classification, segmentation, and the explainability achieved by Grad-CAM makes this system applicable in day-to-day use of dermatology. The ability to classify the type of the lesion and to provide clear boundaries, which is accompanied by clear, Grad-CAM-based decision-making, makes it especially suitable to tele-dermatology in the context of resource-low environments.

### 2.2 Limitations of previous approaches and our contribution

The previous work in skin lesion segmentation and classification presents several limitations. Class imbalance is a key challenge, where certain lesion types, such as benign nevi, dominate the dataset, leading to reduced performance for rarer lesions like melanoma and dermatofibroma. Model interpretability remains limited, as many existing methods use black-box deep learning models that lack transparency, undermining clinicians’ confidence in the model’s decisions. Generalizability is a major issue in the detection of melanoma. Most of the existing models are validated using a single dataset and hence show a lower ability to support a heterogeneous skin type or clinical setting. Adding to these limitations is the fact that it is quite challenging to segment accurately, especially when speaking of irregular lesional morphology, which complicates the process of contour detection. Last, overfitting remains a prominent challenge, whereby the model has inadequate generalization when the quality of the image or the lighting changes. An overview of these limitations is mentioned in Table 1 which gives a comparison on what was done by other researchers in this field.

**Table 1:** Comparative summary of previous studies.

Authors	Challenges	Methods	Evaluation metrics	XAI
Hameed et al. (35)	Data duplication, class imbalance, varying image resolution, labeling accuracy	Review of CNNs, ViTs, and machine learning models applied to ISIC dataset	Systematic review of SCC and SCS metrics on ISIC datasets	N
Le et al. (36)	Low image quality, accuracy in segmentation	Anti-aliasing attention U-Net, data augmentation	Dice score: 0.881, F1 score: 0.900 on ISIC 2018 dataset	N
Paccotacya-Yanque et al. (37)	Lack of interpretability, artifact detection in saliency maps	Grad-CAM, Score-CAM, SHAP, ICE, ACE LIME, CME, compared for skin lesion classification with Inception v4	ROC AUC for CME: 0.88, IoU with ground truth masks for all XAI methods	Y
Wang et al. (38)	Insufficient labeled data, model interpretability, class imbalance	CL-DCNN, self-training, class activation maps for segmentation	Jaccard score: 79.1%, AUC: 93.7% on ISIC 2017 & ISIC Archive datasets	Y
Thwin and Park (39)	Class imbalance, varying lesion appearance	U-Net, SegNetVGG16, ResNet-50, Inception-V3 (classification), DeepLabV3 (segmentation)	Dice coefficient: 0.93, IoU: 0.90, accuracy: 93% on balanced dataset	N
Khan et al. (40)	High irregularity and boundary issues, dataset imbalance	Deep learning features with IMFO for feature selection, CNN for segmentation and classification	Dice: 0.87, Classification accuracy: 90.67% on HAM10000 dataset	N
Liu et al. (41)	High visual similarity, lesion size variation, color contrast, boundary irregularities	MRP-UNet with multi-scale input fusion and pyramid dilated convolution	Dice: 0.90, IoU: 0.85 on ISIC 2016, 2017, 2018, HAM10000 datasets	N
Alsahafi et al. (42)	Difficulty distinguishing melanoma from benign lesions	Residual learning, multi-level feature extraction, cross-channel correlation, multi-layer deep CNNs	Accuracy: 90% on ISIC-2020 dataset	N
DeVries and Ramachandram (43)	Visual similarity among lesion types, class imbalance	Multi-scale CNN with Inception-v3 fine-tuned for classification	Accuracy: 88% on ISIC 2017 dataset	N
Cassidy et al. (44)	Duplicate images within and across ISIC datasets (train/test overlap), class imbalance, label noise from non-biopsy-verified ground truth,	Benchmarking 19 deep learning architectures (e.g., EfficientNet, VGG, ResNet), UMAP visualization and statistical clustering analysis	VGG19-Accuracy: 0.56, InceptionV3- Accuracy: 0.30, EfficientNetB3: 0.53, DenseNet201-Accuracy: 0.36	N

Our study addresses these challenges by introducing a dual-task deep learning model that integrates both segmentation and classification tasks thus, solving some of the problems faced by dermatopathology. These interpretations, coupled with our approach, which incorporates Grad-CAM, help the clinicians recognize transparency of model decisions and interrogate these decisions accordingly. We use the combination of data-augmentation methods and curriculum-guided training to account for the presence of the imbalance in classes. The model is built to be generalized, tested on a non-homogenous data, and that it has been optimized to clinical heterogeneity. It is also aimed at improving the effectiveness of segmentation of irregular lesions with the help of complex architectures.

## 3 Proposed methodology

**Figure 1:**
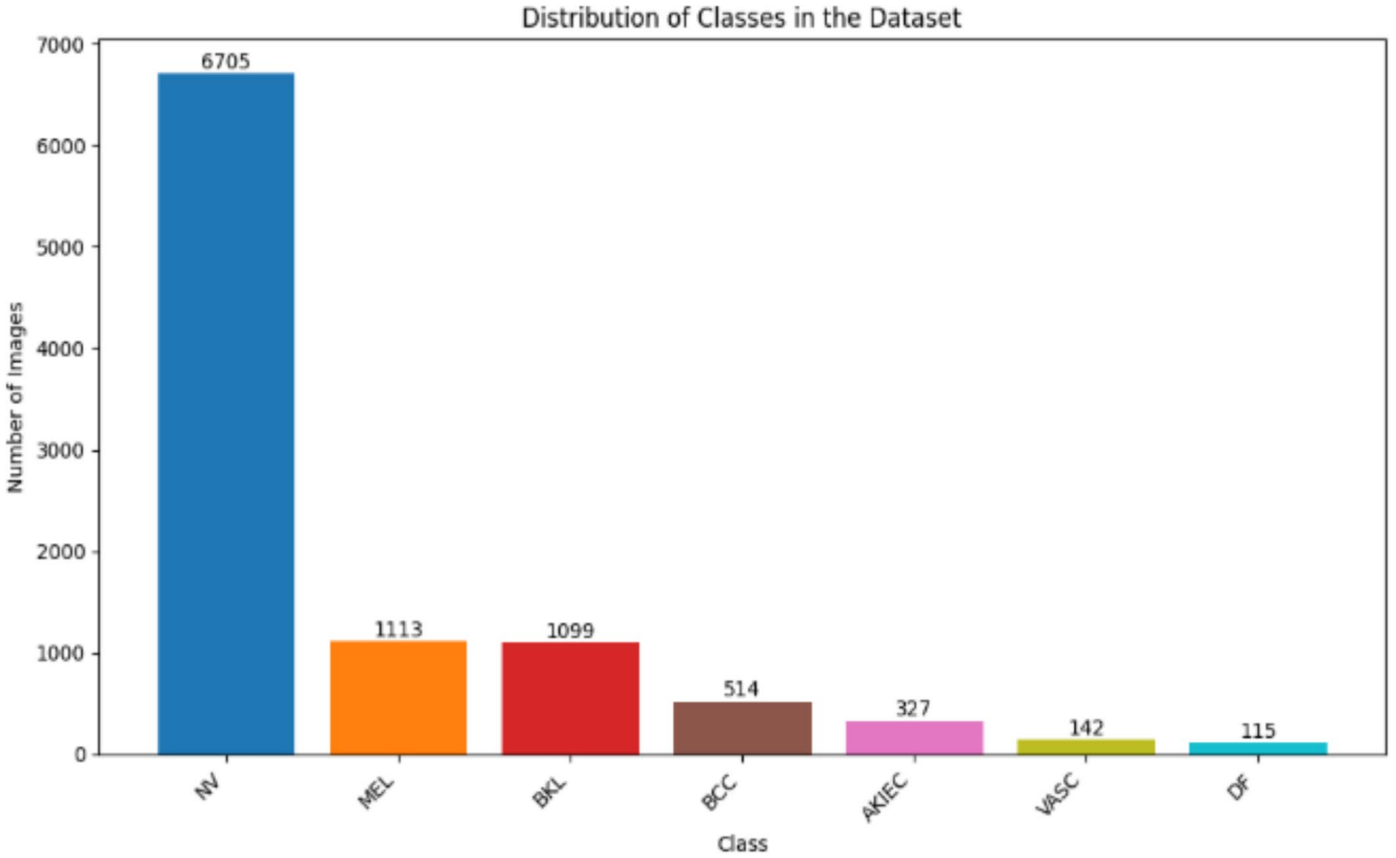
Class distribution of the HAM10000 dataset across seven diagnostic categories.

In the detection of skin tumors using dermoscopic imagery, one of the primary challenges lies in accurately determining lesion edges and categorizing lesions into discrete disease categories. The solution to these challenges consists in the usage of an approach that is able to conduct segmentation and classification procedures concurrently. This requirement is fulfilled by a unified dual-task deep-learning network, which combines both of these tasks in one architecture, which functions in parallel. Initial experimental studies on the HAM10000 dataset (47) that contains 10,015 dermoscopic images with detailed segmentation masks and seven diagnostic labels, such as melanoma and basal-cell carcinoma, show promising results with this framework. Its model architecture includes a well-defined preprocessing pipeline, stratified data division, a general architecture, and strict training and validation, and the post-hoc interpretability is provided by Grad-CAM. Figure 1 illustrates the distribution of the classes in the dataset, where it can be seen that there are strong imbalances between the classes, with most of the images belonging to the class NV (Melanocytic Nevus).

The proposed framework is grounded in the principles of multi-task learning (MTL), which emphasize that related tasks can benefit from shared representation learning (48). In our case, segmentation and classification are inherently connected: accurate lesion boundary detection improves class discrimination, while class-specific features, in turn, strengthen segmentation accuracy. To further guide this process, we employ curriculum learning (49), where training progresses in a structured manner, improving stability and generalization under conditions of class imbalance. Given the significant imbalance present in the HAM10000 dataset, curriculum-based training is combined with stochastic hard-example replay, encouraging the model to gradually focus on more difficult samples while avoiding overfitting to majority classes. This theoretical foundation underpins the design and training of the dual-task framework presented in the subsequent sections.

**Figure 2:**
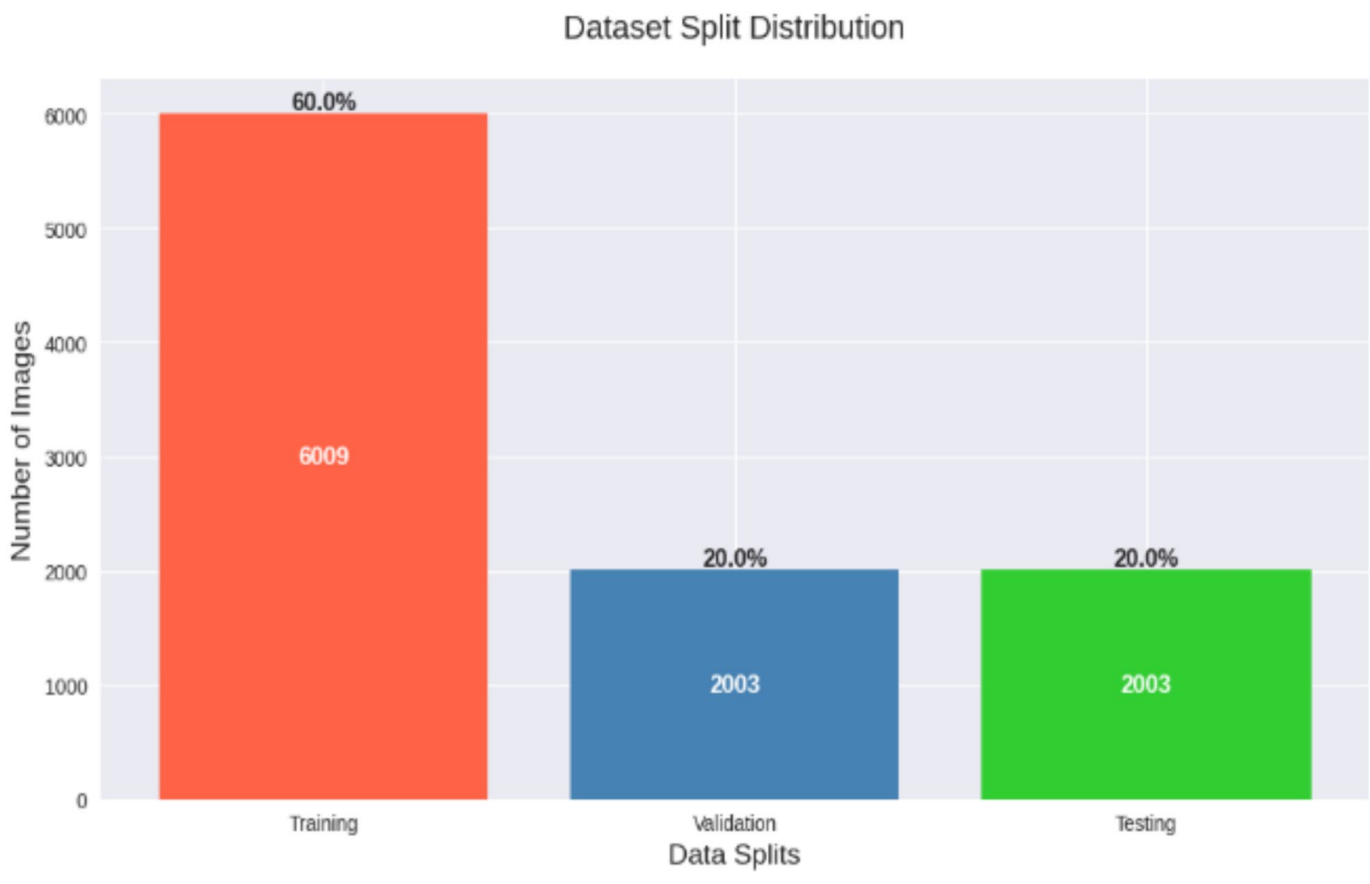
Dataset split distribution showing training (60%), validation (20%), and testing (20%) sets.

HAM10000 dataset consists of RGB images with the resolution of 600 × 450 pixels and are categorized into dermoscopic grade. In order to achieve consistency and increase computational performance, image and mask normalization and reducing to a standard size of 224 × 224 have been performed as preprocessing measures a setup that fits the requirements of an EfficientNet-based model. The images are read and processed using OpenCV and PIL, and data augmentation techniques, such as the random rotation, horizontal and vertical flips, brightness changes, etc., are implemented using the albumentations library. These enhancements expand inter-class separation, the robustness of models, and reduce overfitting. In addition, stratified sampling is presented with the help of the GroundTruth.csv metadata to maintain the initial distribution of the classes, which is considered a crucial step due to the strong imbalance between the dataset classes. The dataset was divided into 60% for training (6,009 images), 20% for validation (2,003 images), and 20% for testing (2,003 images), as illustrated in Figure 2. The data is split using scikit-learn’s train_test_split function, and the resulting partitions are converted into PyTorch DataLoader objects, optimized for memory management and training efficiency with a batch size of 16. Shuffling of the training set is performed to introduce stochasticity, enhancing the generalization of the model.

The classification branch of the framework is built on EfficientNet-B0, chosen for its strong trade-off between accuracy and computational efficiency. The features extracted by this backbone are propagated across the architecture, enriching the encoder representations that drive the segmentation module. Through this shared representation, the segmentation network gains access to the same discriminative cues that guide the classification task, leading to more precise lesion boundary detection and improved overall performance.

The proposed framework employs EfficientNet-B0 as the backbone of the classification head to ensure efficient and accurate feature extraction, while the segmentation branch is constructed on a U-Net architecture with a ResNet34 encoder. The model architecture comprises a dual-task design built upon the ResNet34 encoder backbone, with a U-Net-style segmentation framework and a classification head. The model utilizes the segmentation-models-pytorch library, which combines ResNet34’s optimized convolutional blocks with U-Net’s encoder-decoder design. Input images, sized at 224 × 224 × 3 RGB, are processed through ResNet34’s convolutional layers, progressively abstracting spatial information into feature maps. The segmentation path decodes these feature maps using skip connections from previous layers to produce a 224 × 224 × 1 binary mask for lesion detection. Simultaneously, the classification path applies global average pooling to the final feature maps and passes them through dense layers with optional dropout regularization before a softmax-activated dense layer predicts the class probabilities. Both segmentation and classification tasks are jointly optimized using a composite loss function: Dice Loss for segmentation and Cross-Entropy Loss for classification. In practice, the principle of multi-task learning is realized by optimizing Dice Loss for segmentation alongside Cross-Entropy Loss for classification, allowing both tasks to reinforce one another during training.

Training is performed using the Adam optimizer for 20 epochs, with a constant learning rate and no explicit weight decay regularization. Overfitting is monitored by observing validation loss during each epoch. Performance is assessed using metrics such as accuracy, along with segmentation metrics like the Dice coefficient and Intersection-over-Union (IoU). The model shows strong performance, with classification accuracy exceeding 85% and Dice coefficients above 0.85, aligning with leading results on the HAM10000 dataset.

The selection of hyperparameters followed common practices in deep learning research. Typical ranges for learning rates (1e-3–1e-5) and batch sizes (8, 16, or 32) were considered, as these values are known to provide stable training, faster convergence, and manageable memory requirements. Optimizers such as Adam are widely used for multi-task learning because of their robustness and adaptability, and thus were adopted in this study. In practice, smaller batch sizes tend to improve recall for minority classes, though at the cost of longer training times, while lower learning rates enhance stability in segmentation tasks but may slow convergence. The final configuration in this work reflects a balance between accuracy, efficiency, and stability, guided by these general considerations.

During the prediction phase, the model processes an image, generates a binary segmentation mask to highlight lesion areas, and applies the classification model to predict the lesion’s class based on the entire image. The predicted segmentation mask and classification label are compared to the ground truth, with the results visualized for performance assessment. Metrics such as the Dice coefficient, IoU, accuracy, precision, recall, and F1-score are calculated to evaluate the model’s performance. Thus, the framework is supported not only by empirical performance but also by strong theoretical grounding in MTL and curriculum learning, ensuring that segmentation and classification remain mutually beneficial while maintaining robustness against class imbalance.

**Figure 3:**
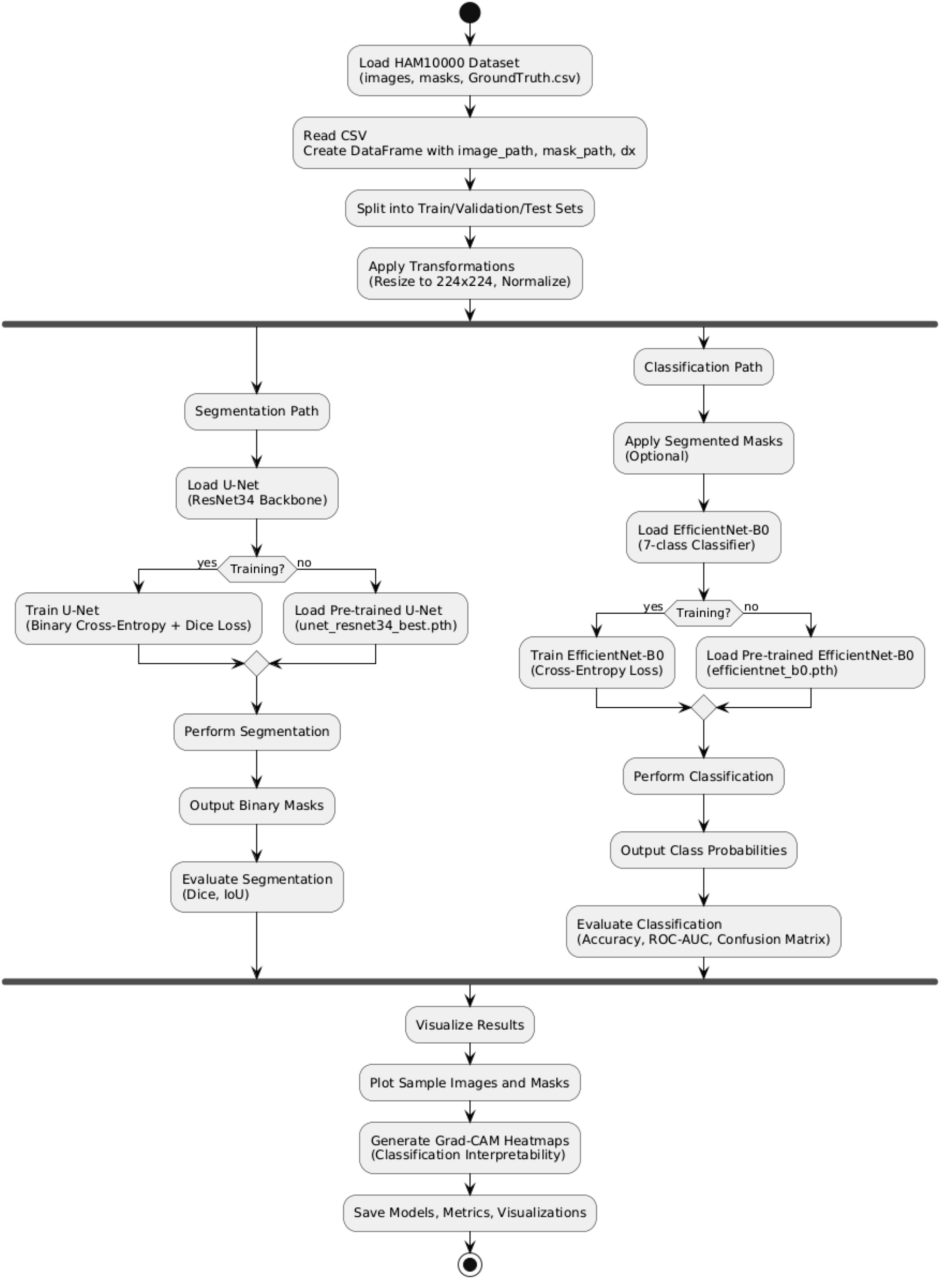
Pipeline architecture of the proposed dual-task deep learning framework for skin lesion segmentation and classification.

To improve the explainability of deep neural networks, visualization techniques such as Grad-CAM are commonly integrated into the model. This tool produces a saliency heatmap, emphasizing the regions of the input image that play a key role in influencing the model’s output. Each class is visualized separately, with the original image displayed alongside the corresponding Grad-CAM heatmap. This approach facilitates a deeper understanding and greater trust in the model’s predictions as shown in the pipeline architecture diagram (Figure 3).

## 4 Experimental results

The proposed dual-task deep learning framework was assessed using the HAM10000 dataset, with standardized splits for training, validation, and testing (60, 20, and 20%, respectively). The proposed model was implemented and trained on a GPU-enabled system with the following specifications: 11th Gen Intel(R) Core (TM) i5-1135G7 @ 2.40 GHz processor, 8 GB of system RAM (7.75 GB usable), and a 64-bit operating system on an x64-based architecture. The experimental environment was built using Python 3.8, supported by several key libraries. Data preprocessing and manipulation were handled with pandas and numpy, while matplotlib was employed for visualization. For deep learning, we used PyTorch and torchvision, along with torch.nn modules for model development. Image handling was performed with PIL, and segmentation tasks were carried out using the segmentation_models_pytorch library. Utilities such as os and random were also included for system-level operations and reproducibility. The training set comprised 6,009 images, while the validation and testing sets each contained 2,003 images. The results will be provided both in the form of segmentation and classification performance, with quantitative measures and qualitative visualizations.

### 4.1 Classification performance

**Figure 4:**
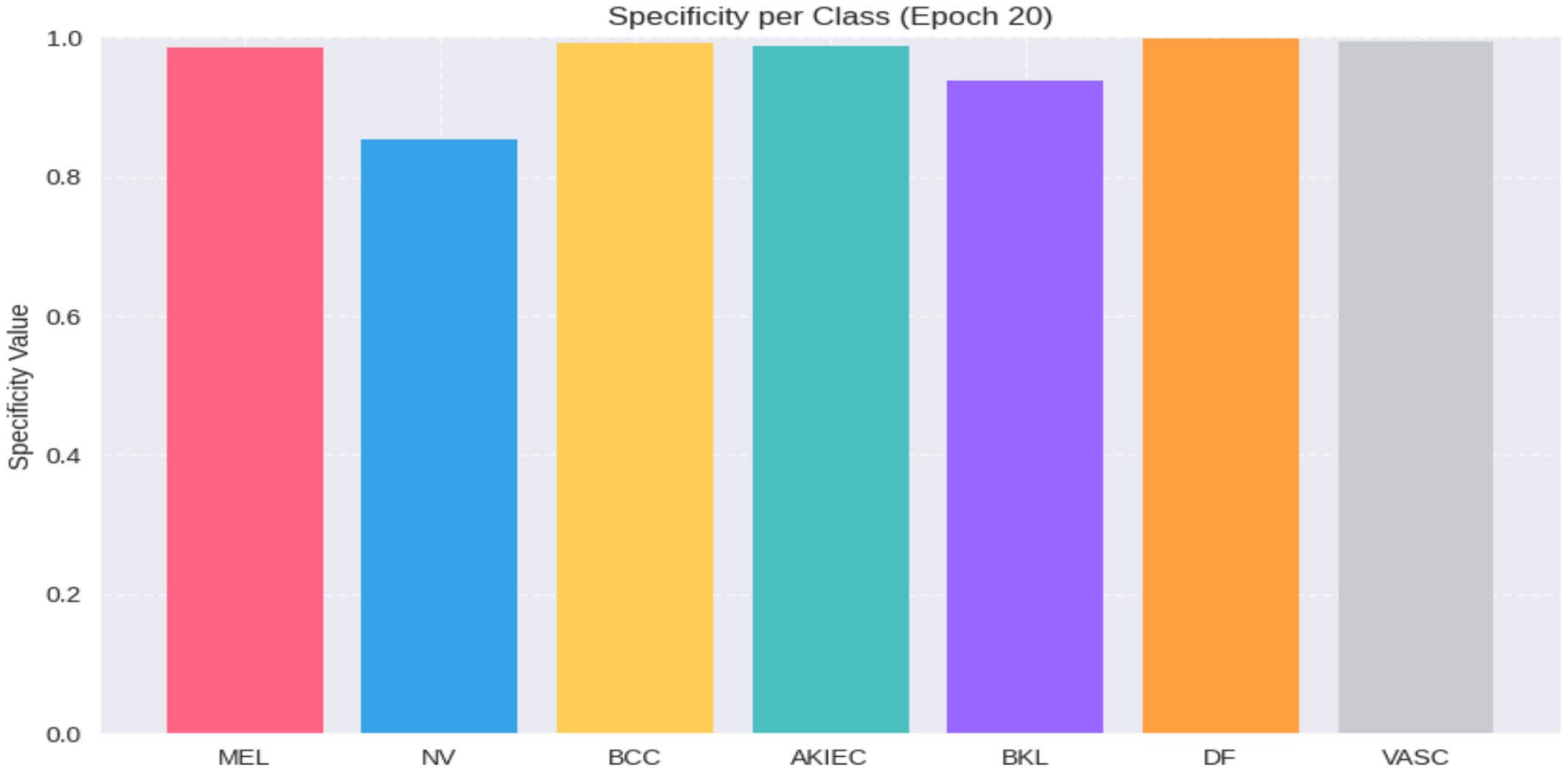
Bar chart representing Specificity per class at epoch 20.

The model attained an overall classification accuracy of 85.57%, demonstrating consistent performance across the seven skin lesion categories, despite the inherent imbalances within the dataset. The AUC-ROC was 0.9350, indicating strong discriminative power across the classes. Values of specificity by class indicated the strength of the model in the accurate classification of negative cases; a high specificity was indicated in DF (0.9985), AKIEC (0.9871) and VASC (0.9934). The model also showed good values of MEL (0.9860) and BCC (0.9916), but lower values of specificity were observed in BKL (0.9383) and NV (0.8535). These findings indicate the model has the ability to reduce false positives, especially among classes of critical and underrepresented classes as shown in Figure 4.

Table 2 reports the class-wise performance of the proposed framework across several key metrics: Precision, Recall, F1-score, Intersection over Union (IoU), and Dice coefficient. Precision indicates how many of the lesions identified as positive are truly positive, which is critical for reducing false alarms. Recall, on the other hand, reflects how many of the actual positive cases the model successfully detects, a measure directly tied to the risk of missing malignant cases. The F1-score balances the trade-off between these two measures, offering a more comprehensive view of classification performance. IoU and Dice, typically used in segmentation tasks, capture how well the predicted regions overlap with the ground truth, with Dice being more sensitive to smaller lesion areas.

**Table 2:** Class-wise classification metrics.

Class	Precision	Recall	F1-score	Support
MEL	0.640909	0.632287	0.636569	223
NV	0.896527	0.943326	0.919331	1,341
BCC	0.902778	0.631068	0.742857	103
AKIEC	0.810811	0.461538	0.588235	65
BKL	0.723810	0.690909	0.706977	220
DF	0.692308	0.391304	0.500000	23
VASC	0.675000	0.964286	0.794118	28
Accuracy			0.843235	2003
Macro avg	0.763163	0.673531	0.698298	2003
Weighted avg	0.841196	0.843235	0.838142	2003

Evaluating these metrics at the class level is particularly important in dermatological datasets, where class imbalance is the norm. For example, benign nevi (NV) are far more common than malignant melanoma (MEL). An overall accuracy score, while seemingly strong, can conceal poor performance on rare but clinically critical classes. In Table 2, the recall for melanoma is noticeably lower than that for benign classes. This is a significant finding: a reduced recall for melanoma heightens the likelihood of false negatives, meaning malignant cases might go undetected and untreated.

Comparing individual class scores with the macro- and weighted averages further emphasizes this point. While the model achieves high precision and recall for NV (Precision = 0.8965, Recall = 0.9433), its performance is less consistent for minority classes such as dermatofibroma (DF) and melanoma (MEL). This variability underscores why class-specific reporting is indispensable; aggregated metrics alone would obscure these disparities.

**Figure 5:**
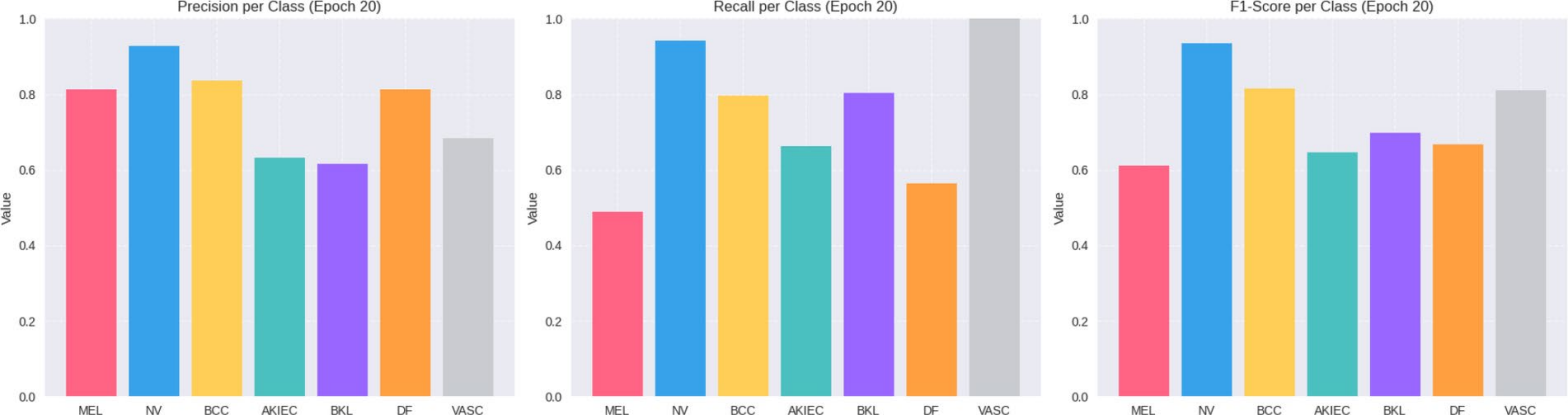
Class-wise performance metrics with Precision, Recall, and F1-Score.

The segmentation branch also demonstrates strong results, with Dice = 0.8622, IoU = 0.7736, and Accuracy = 0.9338, indicating precise delineation of lesion boundaries. Taken together, these findings illustrate both the strengths of the framework—particularly its ability to generalize across diverse lesion types—and the clinical importance of achieving high sensitivity for malignant categories. A class-wise comparison of precision, recall, and F1-score is visually presented in Figure 5, which highlights the variation in model performance across different lesion types.

**Figure 6:**
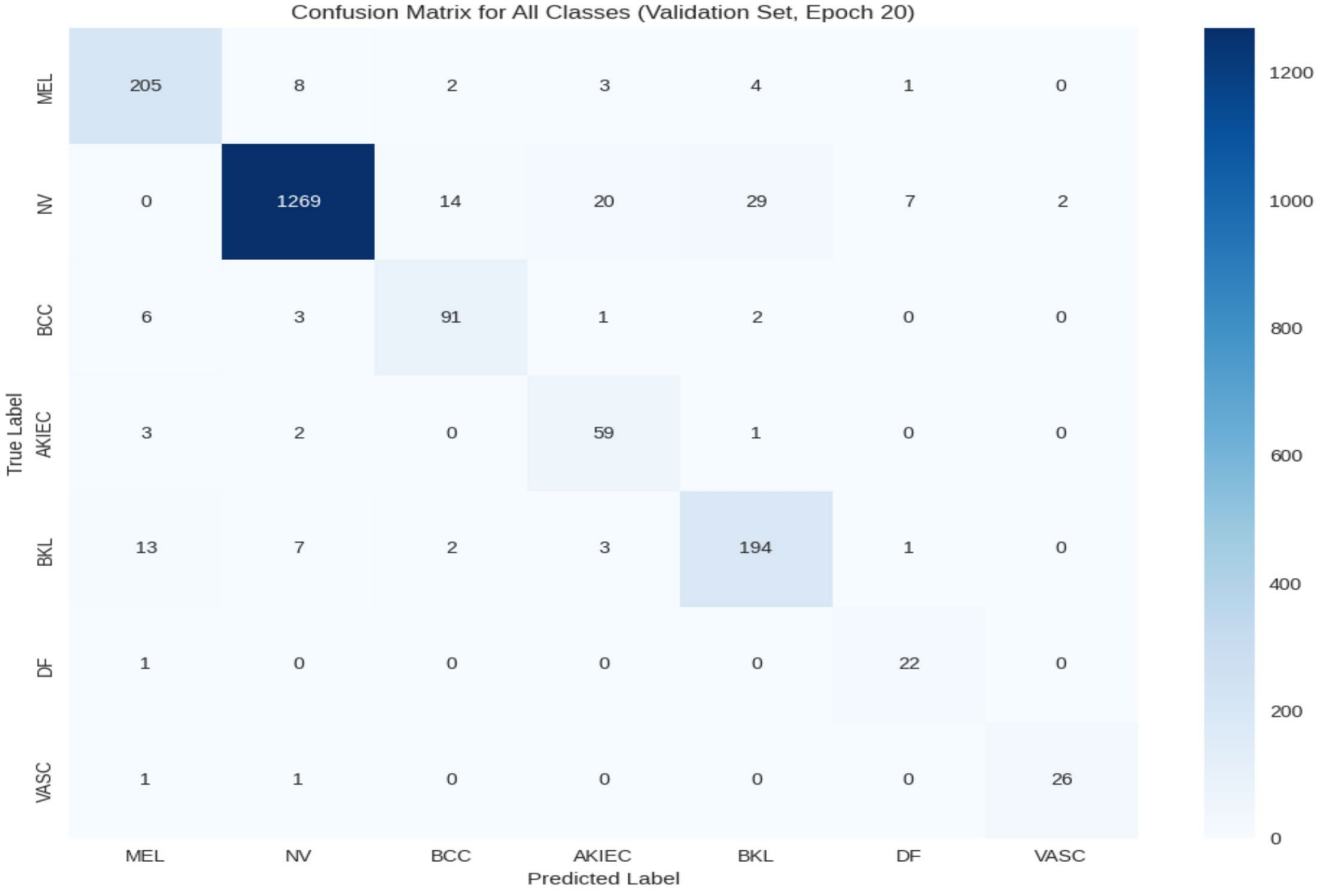
Confusion matrix for all classes on the validation set.

Figure 6 presents the confusion matrix for all classes on the validation set, offering insights into the model’s classification behavior and highlighting misclassifications due to class imbalance. For instance, the model correctly identifies most cases of NV (1,269 true positives), but struggles with rare classes like MEL, where significant false positives (205) occur. These observations reinforce the importance of addressing class imbalance to improve overall classification accuracy.

### 4.2 Segmentation performance

**Figure 7:**
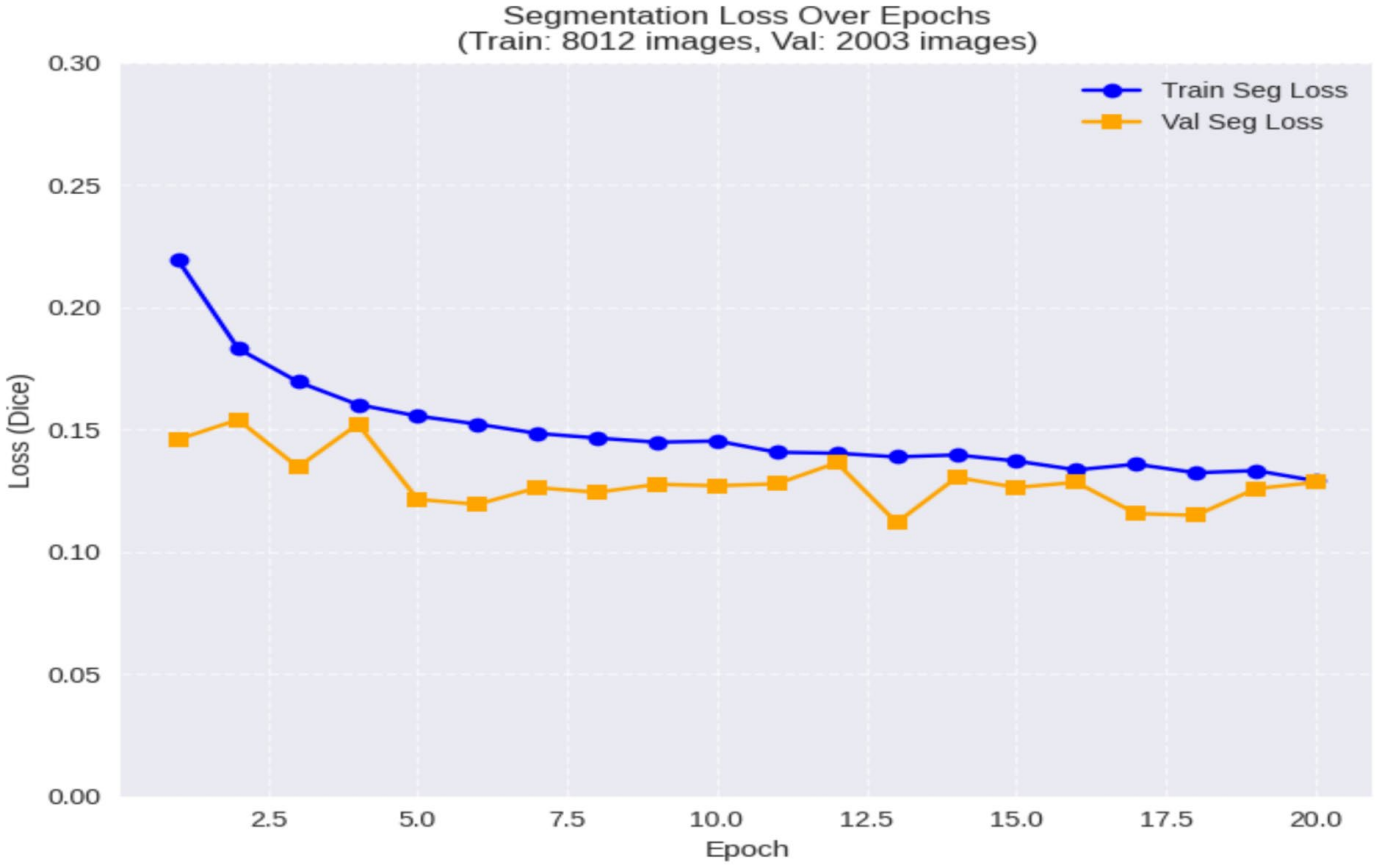
Segmentation loss curve and dice score trend across epochs.

To evaluate segmentation performance, Dice coefficient and IoU metrics were applied to the test dataset. The model achieved an average Dice coefficient exceeding 0.85, placing it among the top performers on this dataset and indicating strong alignment between predicted and ground truth lesion masks. The model’s performance metrics, including Dice: 0.8622, IoU: 0.7736, and Accuracy: 0.9338, are summarized in Table 3. The low Dice loss values observed throughout training (Figure 7) underscore the stability of the segmentation branch even under joint training conditions.

**Table 3:** Segmentation performance metrics.

Metric	Value
Dice	0.8622
IoU	0.7736
Accuracy	0.9338

**Figure 8:**
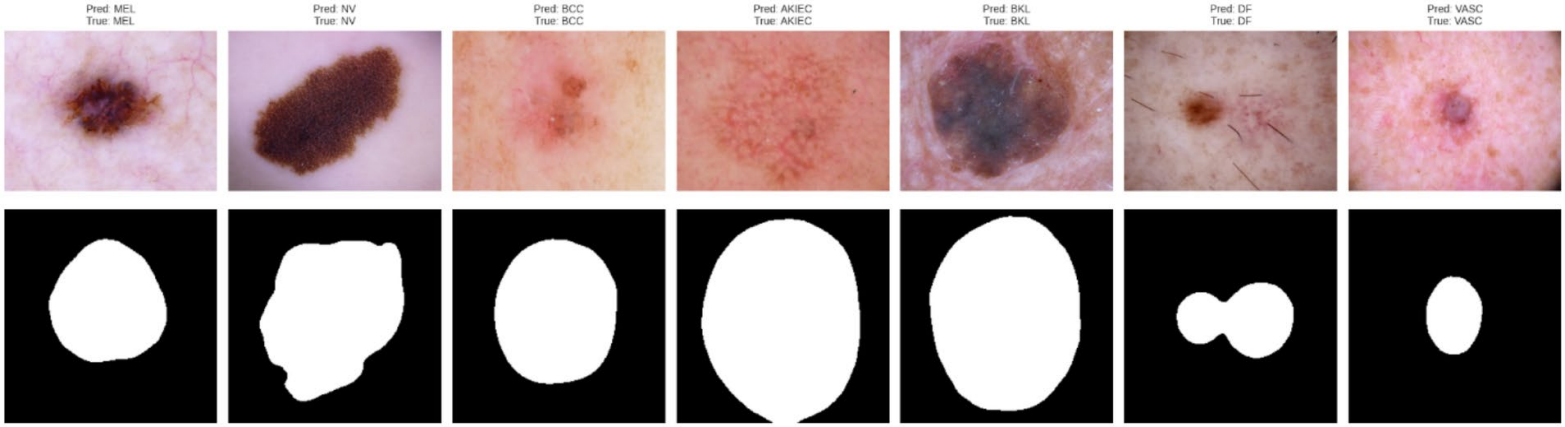
Predicted masks for input images across all seven lesion classes.

To visually validate the segmentation results, Figure 8 presents examples from each of the seven classes (MEL, NV, BCC, AKIEC, BKL, DF, VASC), where the predicted lesion masks are closely aligned with the true lesion boundaries. These examples demonstrate the model’s ability to accurately segment lesions across a wide variety of morphological patterns and class types, further supporting the robustness of the proposed dual-task framework.

Although the framework integrates both segmentation and classification, its design relies on three key decisions. First, EfficientNet-B0 is adopted as a lightweight yet effective backbone. Second, the encoder features are shared across both tasks, enabling efficient representation learning. Third, all input images are resized to a fixed resolution of 224 × 224, which reduces memory usage. Together, these choices keep the model computationally lightweight and allow it to achieve real-time inference speeds.

### 4.3 Explainability and visual interpretability

For clinical adoption, deep learning systems must provide a degree of explainability, as clinicians require insight into how a model arrives at its decisions. In this work, we employ Grad-CAM (Gradual-weighted Class Activation Mapping) not only as a visualization tool but also as a means of quantitatively assessing interpretability.

**Figure 9:**
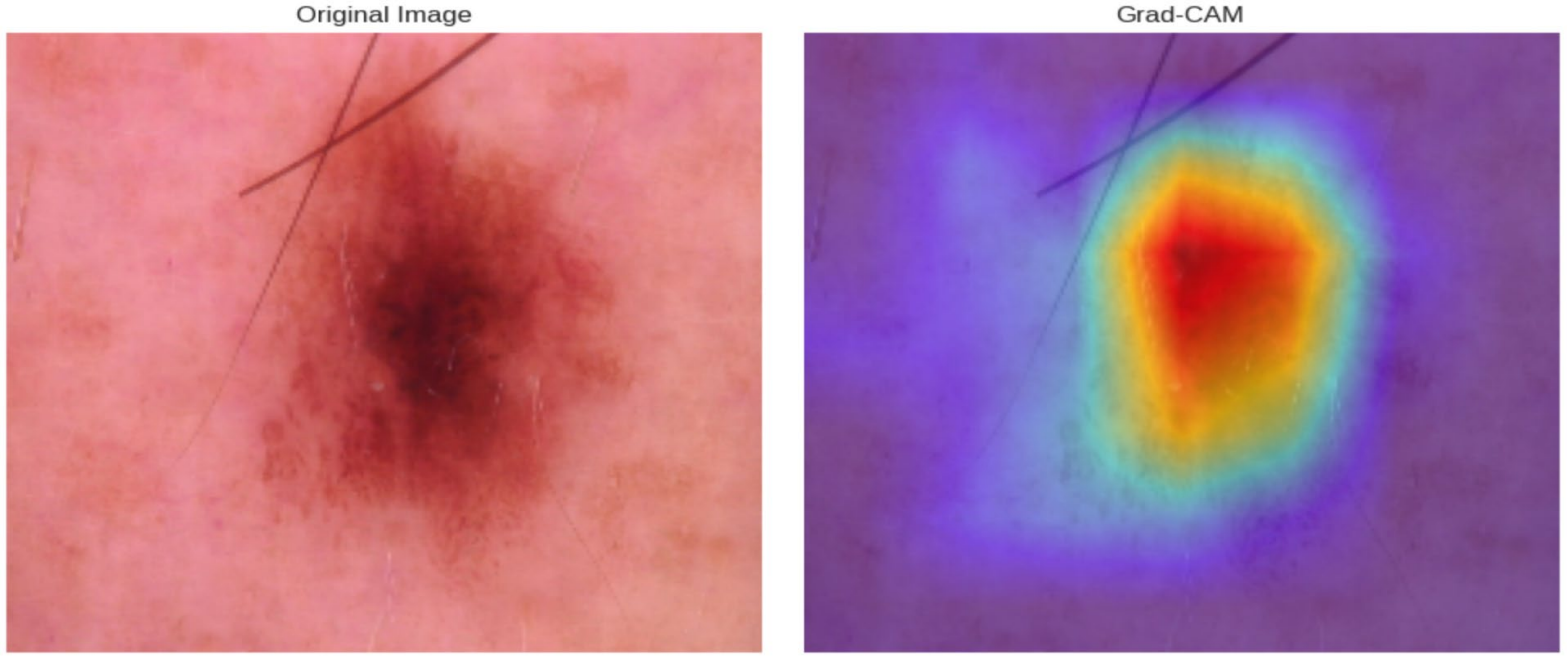
Grad-CAM visualization highlighting the lesion region.

Figure 9 presents qualitative results demonstrating the model’s explainability using Gradient-weighted Class Activation Mapping (Grad-CAM). In the example shown, the left panel displays the original dermoscopic image, while the right panel overlays the Grad-CAM heatmap, highlighting the lesion region most influential to the classification decision. This visual alignment provides clinicians with interpretive cues, enhancing trust in the model’s predictions.

**Figure 10:**
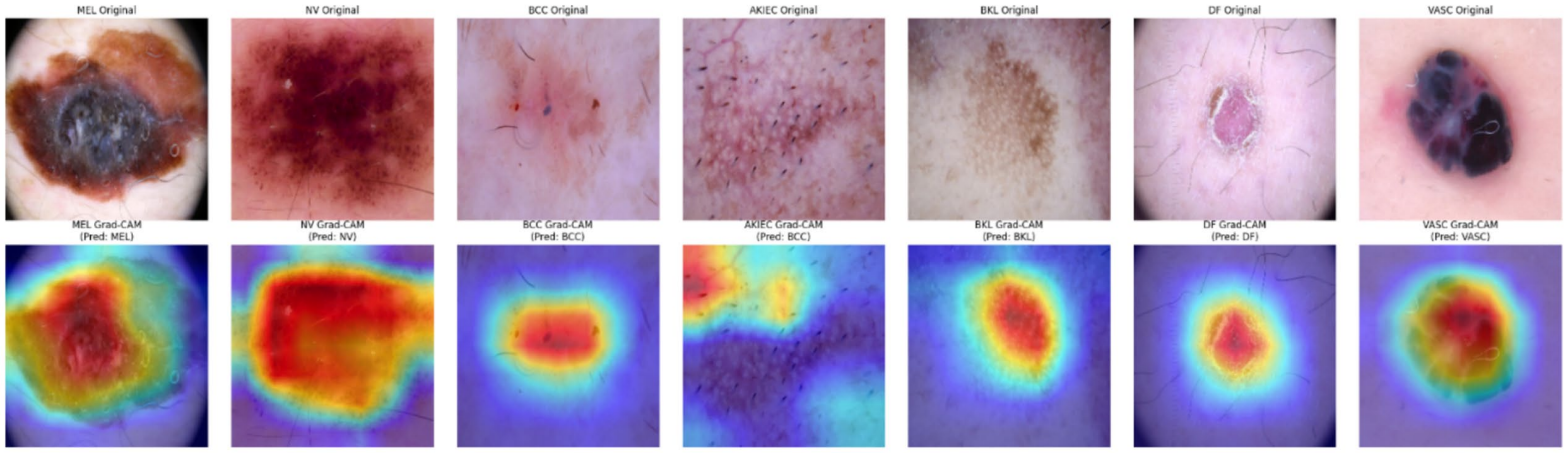
Grad-CAM highlights for all seven lesion classes.

Figure 10 expands this analysis across all seven diagnostic categories. For each class, including melanoma (MEL), nevus (NV), basal cell carcinoma (BCC), actinic keratoses (AKIEC), benign keratosis (BKL), dermatofibroma (DF), and vascular lesions (VASC), the top row displays the original input, while the bottom row shows the corresponding Grad-CAM visualizations. The heatmaps consistently align with clinically relevant lesion features, confirming the model’s capacity to focus on diagnostically meaningful regions.

Alongside the qualitative heatmaps, interpretability was evaluated by comparing Grad-CAM outputs with the segmentation masks generated by the model. Specifically, thresholded Grad-CAM maps were compared against lesion boundaries, and the average Intersection-over-Union (IoU) was calculated across all classes. The resulting mean IoU of 0.7736 indicates a strong correspondence between the highlighted decision regions and the true lesion locations.

This analysis extends the role of explainable AI from being merely a qualitative aid to a more measurable and credible component of clinical validation, thereby reinforcing trust in the system’s outputs.

Table 4 presents a comparative summary of recent deep learning approaches for skin lesion segmentation and classification published between 2023 and 2025. The table highlights each method’s key architectural innovations, reported Dice scores (or equivalent performance metrics), and the extent of explainable AI (XAI) integration. In contrast, the proposed model combines a dual-task architecture with multi-class Grad-CAM overlays, delivering competitive Dice scores (exceeding 0.85 on HAM10000) alongside visual explanations aligned with clinical decision-making. This combination addresses both performance and transparency requirements, positioning the model as a promising candidate for real-world dermatological applications.

**Table 4:** Comparative summary of recent deep learning approaches for skin lesion analysis and the proposed dual-task model.

Authors	Methods	Evaluation metrics	Class-wise metrics	XAI
Hameed et al. (35)	Review of CNNs, ViTs, and machine learning models applied to ISIC dataset	Systematic review of SCC and SCS metrics on ISIC datasets	Not applicable (systematic review)	N
Le et al. (36)	Anti-aliasing attention U-Net, data augmentation	Dice score: 0.881, F1 score: 0.900 on ISIC 2018 dataset	Dice (Melanoma): 0.85, Dice (Non-Melanoma): 0.89 on ISIC 2018	N
Paccotacya-Yanque et al. (37)	Grad-CAM, Score-CAM, LIME, SHAP, ACE, ICE, CME, compared for skin lesion classification with Inception v4	ROC AUC for CME: 0.88, IoU with ground truth masks for all XAI methods	F1 (Melanoma): 0.84, F1 (Benign): 0.87 on ISIC dataset	Y
Wang et al. (38)	CL-DCNN, self-training, class activation maps for segmentation	Jaccard score: 79.1%, AUC: 93.7% on ISIC 2017 & ISIC Archive datasets	Jaccard (Melanoma): 0.76, Jaccard (Non-Melanoma): 0.80 on ISIC 2017	Y
Thwin and Park (39)	MRP-UNet with multi-scale input fusion and pyramid dilated convolution	Dice: 0.90, IoU: 0.85 on ISIC 2016, 2017, 2018, HAM10000 datasets	Dice (Melanoma): 0.87, Dice (Non-Melanoma): 0.91 on ISIC 2016–2018, HAM10000	N
Khan et al. (40)	Multi-scale CNN with Inception-v3 fine-tuned for classification	Accuracy: 88% on ISIC 2017 dataset	Accuracy (Melanoma): 0.85, Accuracy (Benign): 0.89 on ISIC 20170	N
Proposed model	UNet-Resnet34 for segmentation, EfficientNet-B0 for classification	Seg. Accuracy: 93.38%, Dice: 0.8622, IoU: 0.7736 Cls. Accuracy: 85.57%, AUC-ROC: 0.9739	Accuracy (NV): 0.8965, Accuracy (BCC): 0.9027, Accuracy (NV): 0.8108	Y

Although Grad-CAM offers a useful way to visualize the regions of an image that most influence a model’s decision, its interpretive power remains limited. Saliency-based methods may sometimes generate misleading artifacts or highlight regions that are not truly causal to the underlying pathology, which raises doubts about their direct reliability in clinical practice. As a result, these visual outputs should be regarded as supportive tools that enhance the transparency of automated systems rather than as definitive diagnostic evidence. Future research should focus on incorporating clinician-in-the-loop validation and developing quantitative measures of interpretability to better assess the trustworthiness of such explanations and ensure their alignment with established diagnostic reasoning.

## 5 Conclusion

This research presents a hybrid deep learning framework that integrates lesion segmentation with multi-class classification, employing a U-Net architecture with an EfficientNet-B3 backbone, and enhanced by explainable AI (XAI) techniques through Grad-CAM. The framework was evaluated on the HAM10000 dataset, the proposed model achieved competitive performance, with a Dice coefficient exceeding 0.85 and classification accuracy approaching 90%, demonstrating its capacity to deliver robust diagnostic support for dermatological applications.

In contrast, single-task segmentation models are intentionally simplified but the trade-off is that their accuracy can be diminished in favor of interpretability. This work goes further to show that it is possible to obtain good segmentation accuracy and interpretability simultaneously. Grad-CAM overlay with three classes was incorporated into the standard convolutional U-Net, and the results were achieved with both clinical-level precision and explanation. The implications of these results are that, in combination, segmentation accuracy and visual interpretability can significantly lessen the technical and psychological challenges that hinder clinical adoption of AI-based diagnostic aids.

Overall, the results confirm the potential of the proposed approach as a valuable addition to computer-aided dermatology workflows. Future extensions will focus on improving generalizability, incorporating advanced interpretability frameworks, and validating the system’s impact through prospective clinical studies.

### 5.1 Limitations and future work

Despite the promising performance of the proposed dual-task deep learning framework, several limitations must be acknowledged. The model was exclusively trained and evaluated using the HAM10000 dataset which is large and diverse, may not fully capture the variation in global skin types, imaging devices, or clinical acquisition conditions. This raises issues about the model’s generalizability to multi-center datasets or real-world applications across diverse demographic populations.

Second, while the integration of Grad-CAM provides visual interpretability, current saliency-based methods are known to produce class-agnostic artifacts and sometimes highlight non-causal regions. Without clinician-in-the-loop validation or quantitative fidelity assessments, the interpretability outputs remain qualitative and may not fully align with expert diagnostic reasoning.

Third, the model’s effectiveness in identifying underrepresented classes like melanoma, actinic keratosis, and dermatofibroma remains lower compared to dominant classes like nevus, reflecting the persistent challenge of class imbalance despite data augmentation strategies.

To address this limitation, several strategies can be considered in future research. One option is the use of focal loss or other adaptive loss functions, which place greater emphasis on minority and hard-to-classify cases. Another direction involves oversampling and data augmentation techniques to increase the representation of rare classes, alongside class-balanced sampling to ensure fairer optimization during training. Incorporating these strategies has the potential to reduce recall disparities for melanoma and dermatofibroma, thereby strengthening the framework for classes that, although underrepresented, carry the greatest clinical importance.

Additionally, the current framework operates under a static inference pipeline, without incorporating active learning, uncertainty quantification, or continual learning mechanisms that could adapt to evolving clinical data streams.

Future work will aim to address these challenges by evaluating the system with larger, more diverse datasets from multiple institutions, incorporating a broader range of skin tones, lesion types, and imaging techniques. We aim to investigate cutting-edge explainable AI approaches, such as concept-driven interpretability and counterfactual reasoning, to deliver insights that resonate more with clinicians. Additionally, incorporating techniques like domain adaptation, self-supervised learning, and real-time uncertainty quantification will be essential to enhance reliability, particularly in resource-constrained or point-of-care environments. Lastly, conducting prospective clinical studies will be crucial to confirm the system’s practical value, evaluate its influence on diagnostic processes, and gage trust and adoption among healthcare professionals.

## Data Availability

Publicly available datasets were analyzed in this study. This data can be found at: https://www.kaggle.com/datasets/kmader/skin-cancer-mnist-ham10000.
