# Correction: An explainable hybrid deep learning framework for precise skin lesion segmentation and multi-class classification

**DOI:** 10.3389/fmed.2025.1724427

**Published:** 2025-10-29

**Authors:** Muhammad Fiaz, Muhammad Bilal Shoaib Khan, Abdul Hannan Khan, Anas Bilal, Monir Abdullah, Abdulbasit A. Darem, Raheem Sarwar

**Affiliations:** ^1^Department of Computer Science, Green International University, Lahore, Pakistan; ^2^Department of Software Engineering, University of Central Punjab, Lahore, Pakistan; ^3^College of Information Science and Technology, Hainan Normal University, Haikou, China; ^4^Department of Computer Science and Artificial Intelligence, College of Computing and Information Technology, University of Bisha, Bisha, Saudi Arabia; ^5^Center for Scientific Research and Entrepreneurship, Northern Border University, Arar, Saudi Arabia; ^6^Department of Computer Science, College of Science, Northern Border University, Arar, Saudi Arabia; ^7^OTEHM, Manchester Metropolitan University, Manchester, United Kingdom

**Keywords:** skin disease, classification, segmentation, explainable AI, Grad-CAM

In the published article, there was a mistake in the **Acknowledgments**. The project number for Northern Border University support was shown as “NBU-CRP-2025-2903”. The correct statement is:

“The authors are thankful to the Deanship of Graduate Studies and Scientific Research at the University of Bisha for supporting this work through the Fast-Track Research Support Program, and the authors extend their appreciation to Northern Border University, Saudi Arabia, for supporting this work through project number NBU-FFR-2025-2903-17.”

